# No hypoperfusion is produced in the epicardium during application of myocardial topical negative pressure in a porcine model

**DOI:** 10.1186/1749-8090-2-53

**Published:** 2007-12-06

**Authors:** Sandra Lindstedt, Malin Malmsjö, Richard Ingemansson

**Affiliations:** 1 Department of Cardiothoracic Surgery, Lund University Hospital, Lund, Sweden; 2 Department of Medicine, Lund University Hospital, Lund, Sweden

## Abstract

**Background:**

Topical negative pressure (TNP), commonly used in wound therapy, has been shown to increase blood flow and stimulate angiogenesis in skeletal muscle. We have previously shown that a myocardial TNP of -50 mmHg significantly increases microvascular blood flow in the myocardium. When TPN is used in wound therapy (on skeletal and subcutaneous tissue) a zone of relative hypoperfusion is seen close to the wound edge. Hypoperfusion induced by TNP is thought to depend on tissue density, distance from the negative pressure source, and the amount negative pressure applied. When applying TNP to the myocardium, a significant, long-standing zone of hypoperfusion could theoretically cause ischemia, and negative effects on the myocardium. The current study was designed to elucidate whether hypoperfusion was produced during myocardial TNP.

**Methods:**

Six pigs underwent median sternotomy. Laser Doppler probes were inserted horizontally into the heart muscle in the LAD area, at depths of approximately, 1–2 mm. The microvascular blood flow was measured before and after the application of a TNP. Analyses were performed before left anterior descending artery (LAD) occlusion (normal myocardium) and after 20 minutes of LAD occlusion (ischemic myocardium).

**Results:**

A TNP of -50 mmHg induced a significant increase in microvascular blood flow in normal myocardium (**p = 0.01), while -125 mmHg did not significantly alter the microvascular blood flow. In ischemic myocardium a TNP of -50 mmHg induced a significant increase in microvascular blood flow (*p = 0.04), while -125 mmHg did not significantly alter the microvascular blood flow.

**Conclusion:**

No hypoperfusion could be observed in the epicardium in neither normal nor ischemic myocardium during myocardial TNP.

## Introduction

Vacuum therapy, or topical negative pressure (TNP), can be regarded as an established clinical routine for wound care, in use since the mid or late 1990's, and it has been shown to promote the healing of chronic and problematic wounds [1-3], for example, post-sternotomy mediastinitis[4,5]. The physiological and molecular biological mechanisms by which TNP accelerates wound healing are to a large extent unknown. However, TNP is known to increase the blood flow as a result of mechanical stress and a pressure gradient across the tissue, and increased blood flow is known to stimulate granulation tissue formation, and angiogenesis in subcutaneous tissue and skeletal muscle [6-10].

Mediastinitis is a strong predictor for poor long-term survival after coronary artery by-pass grafting (CABG), when using conventional wound healing techniques (closed irrigation, delayed wound closure, or reconstructing with omentum or pectoral flaps) [11-16]. Recently, the use of TNP has gained acceptance in the treatment of post-sternotomy mediastinitis, and is today the standard mode of treatment in many cardiac surgery centers [4,5,17,18].

Previously, we have showed no difference in long-term survival between CABG patients with TNP-treated mediastinitis and CABG patients without mediastinitis [17]. It may be that the TNP stimulation of blood flow and development of collateral blood vessels in part account for the reduced long-term mortality.

Patients with ungraftable coronary disease may benefit from methods that increase blood flow to the area and stimulate myocardial angiogenesis that are not dependent on vessel caliber. Vascular endothelial growth factor (VEGF) has been found to be one of the most interesting growth factors in therapeutic angiogenesis [19-24]. Interestingly, the mechanical forces exerted by TNP stimulate the endogenous production of VEGF[25,26].

We have previously shown that a myocardial TNP of -50 mmHg significantly increases microvascular blood flow in the underlying myocardium at a depth of 6–8 mm in porcine myocardium[27]. We have also shown that TNP produces relative hypoperfusion close to the wound edge in muscular and subcutaneous tissue[8]. Hypoperfusion in myocardial TNP could theoretically lead to ischemia, with negative consequences in the myocardium. The present study was designed to elucidate whether a relative hypoperfusion zone is produced in the epicardium, at a depth of 1–2 mm in the heart muscle, during the application of TNP.

The microvascular blood flow was measured using laser Doppler velocimetry in a porcine model. The effect of a TNP of -50 and -125 mmHg was investigated before and during occlusion of the left anterior descending artery (LAD) to imitate ischemic coronary disease. No such study has to our knowledge been performed previously.

## Methods

### Experimental animals

A porcine model was use in the present study. Six domestic landrace pigs of both genders, with a mean body weight of 70 kg, were fasted overnight with free access to water. The study was approved by the Ethics Committee for Animal Research, Lund University, Sweden. The investigation complied with the "Guide for the Care and Use of Laboratory Animals" as recommended by the U.S. National Institutes of Health, and published by the National Academies Press (1996).

### Anesthesia

All animals were pre-medicated intramuscularly with ketamine (30 mg/kg) before they were brought into the laboratory. Before commencing surgery sodium thiopental (5 mg/kg), atropine (0.02 mg/kg) and pancuronium (0.5 mg/kg) were given intravenously. Tracheotomy was performed with a Portex endo-tracheal tube (7.5 mm internal diameter, Medcompare™, USA). A servo-ventilator (Siemens Elema 300A, Stockholm, Sweden) was used for mechanical ventilation throughout the experiment. The ventilator settings used were: minute volume = 100 ml/kg, FiO_2 _= 0.5, breathing frequency = 16 breaths/minute and positive end expiratory pressure = 5 cmH_2_O.

Anesthesia and muscular paralysis were maintained with a continuous intravenous infusion of 8–10 mg/kg/hour propofol (Diprivan^®^, AstraZeneca, Sweden), 0.15 mg/kg/hour fentanyl (Leptanal^®^, Lilly, France), and 0.6 mg/kg/hour pancuronium (Pavulon^®^, Organon Teknika, Boxtel, the Netherlands).

### Data acquisition

Mean arterial pressure, central venous pressure, heart frequency, and ventilatory parameters were recorded throughout the experiments.

### Surgical procedure

Surgery was performed through median sternotomy. After heparinization (400 IU/kg) a cardiopulmonary bypass (CPB) was installed with an arterial cannula (22 French, DLP ^® ^Elongated One-Piece Arterial Cannula (EOPA™), Medtronic Inc., Minneapolis, MO, USA) in the distal ascending aorta, and a venous cannula (32 French, MC2^® ^Two-Stage Venous Cannula, also from Medtronic Inc.) inserted through the right atrium. Before cannulation of the heart the cannulae were inserted through the thoracic wall to prevent air leakage during TNP application. CPB was conducted in normothermia. Ventricular fibrillation was subsequently induced in the heart. No aortic cross-clamping was performed and no cardioplegia was employed. The mean arterial pressure was maintained between 60 and 80 mmHg. A left ventricular vent (DLP^® ^Vent, also from Medtronic Inc.) was used to protect the left chamber from overloading. Pulmonary ventilation was applied at a rate of 4 liters/minute during the experiments.

A CPB was used to facilitate the measurements of microvascular blood flow using laser Doppler velocimetry. Fibrillation of the heart minimizes the movement artifacts, while the physiological conditions are, to a large extent, conserved. Moreover, CPB prevents the risk of circulatory failure during LAD occlusion, thereby facilitating experimental analysis in the case of the ischemic myocardium.

Microvascular blood flow was measured using laser Doppler velocimetry (Transonic^® ^Laser Doppler Monitor, BLF21, Maastricht, the Netherlands, and Peri Flux System 5000, Perimed, Stockholm, Sweden), employing atechnique that quantifies the sum of the motion of the red blood cells in a specific volume. This method is extensively applied in plastic surgery procedures and employs a fiberoptic probe carrying a beam of light. Light impinging on cells in motion undergoes a change in wavelength (Doppler shift) while light impinging on static objects remains unchanged. The magnitude and frequency distributions of the changes are directly related to the number and velocity of red blood cells. The information is collected by a returning fiber, converted into an electronic signal, and analyzed[28].

Laser Doppler probes were inserted horizontally into the heart muscle 6–8 mm lateral of the LAD at depths of approximately 1–2 mm. All probes were carefully fixed to the surface of the heart with a suture (Prolene 7-0; Ethicon Inc., New Jersey, USA), thereby preventing probe movement. After the experiments, the heart was dissected and the probe location was confirmed. A round hole, 5 cm in diameter, was made in the middle of a phrenic nerve pad (Phrenic Nerve Pad^® ^Medtronic Inc.) and placed on top of the heart. The pad was stabilized to the surrounding myocardium by 8–10 sutures (Prolene 5-0; Ethicon Inc., New Jersey, USA) and by sutures to the posterior sternal edges (Dermalon 2-0; Davis and Geck, St. Louis, New Jersey, USA). A retractor was used throughout the experiments to keep the sternal edges apart. A polyurethane foam dressing, with an open pore structure of 400 to 600 μm (KCI, Copenhagen, Denmark) was placed between the sternal edges. The foam was continuously sutured to the surrounding skin (Dermalon 2-0; Davis and Geck). The wound was sealed with a transparent adhesive drape. A Track Pad (KCI, Copenhagen, Denmark) was inserted through the drape and was connected to a vacuum pump, (V.A.C. pump unit, KCI, Copenhagen, Denmark). When the negative pressure is applied, the heart will be drawn up towards the phrenic nerve pad and the foam without interfering with the sternal edges. This procedure causes the application of negative pressure to affect only the myocardium exposed through the 5 cm diameter hole.

### Experimental protocol

The microvascular blood flow was measured continuously by the laser Doppler filament probes. Recordings were made in normal myocardium, before negative pressure was applied, and at negative pressures of -50 and -125 mmHg.

The LAD was then occluded for 20 minutes with an elastic vessel loop. Microvascular blood flow was measured before, and after 5, 10, 15 and 20 minutes of occlusion.

Recordings were also made in ischemic myocardium, before negative pressure was applied, and at negative pressures of -50 and -125 mmHg.

### Calculations and statistics

Laser Doppler velocimetry measurements were performed on six pigs. The output was continuously recorded using PeriSoft software (Perimed, Stockholm, Sweden). Microvascular blood flow was expressed in terms of perfusion units (PU). Calculations and statistical analysis were performed using GraphPad 4.0 software. Statistical analysis was performed using Student's paired t-test. Significance was defined as *p < 0.05, **p < 0.01, ***p < 0.001 and p > 0.05 (not significant, n.s.). Values are presented as means ± the standard error on the mean (SEM).

## Results

### Normal myocardium

**Figure 1:**
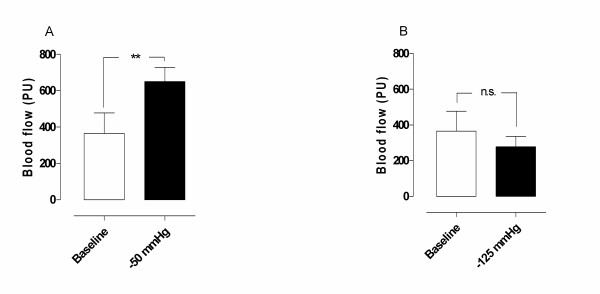
Microvascular blood flow measured using laser Doppler velocimetry in normal myocardium exposed to topical negative pressures of A) -50 mmHg, and B) -125 mmHg. The measurements were performed in six pigs at a depth of 1–2 mm in the myocardium. Significance was defined as *p < 0.05, **p < 0.01, ***p < 0.001 and p > 0.05 (not significant, n.s.). Values are presented as means ± the standard error on the mean (SEM).

A topical negative pressure of -50 mmHg induced an immediate significant increase in microvascular blood flow in normal myocardium (from 365.8 ± 111.7 PU before, to 649.8 ± 78.3 PU after TNP application, ** *p *= 0.01) (Figure 1A). A TNP of -125 mmHg did not result in any significantly change in microvascular blood flow (365.8 ± 111.7 PU before, and 279.2 ± 57.1 PU after TNP application, *p *= 0.33) (Figure 1B).

### LAD occlusion

**Figure 2:**
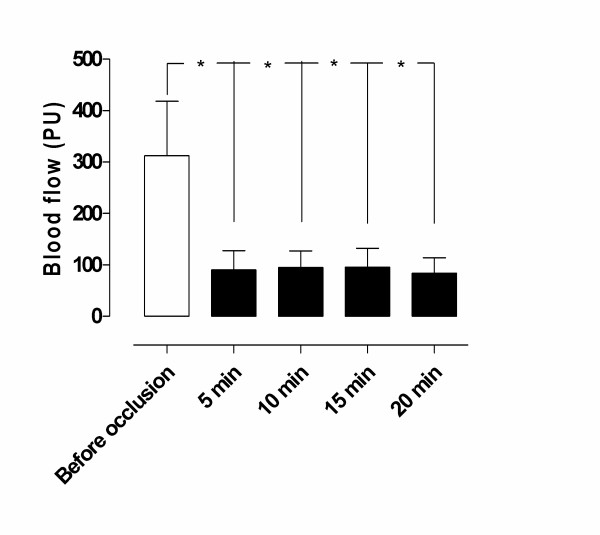
Microvascular blood flow measured using laser Doppler velocimetry in the myocardium before and after 5, 10, 15, and 20 minutes of occlusion of the left anterior descending artery (LAD). Note the decrease in microvascular blood flow, from 311.7 ± 106.2 PU before, to 84.2 ± 29.9 PU after 20 minutes' occlusion of the LAD, in the area studied. Significance was defined as *p < 0.05, **p < 0.01, ***p < 0.001 and p > 0.05 (not significant, n.s.). Values are presented as means ± the standard error on the mean (SEM).

Ischemia was induced by occlusion of the LAD for 20 minutes. The blood flow was 311.7 ± 106.2 PU before occlusion of the LAD, and decreased to 90.0 ± 37.3 PU after 5 minutes (*p = 0.03), to 95.0 ± 32.2 PU after 10 minutes (*p = 0.04), to 95.8 ± 36.9 after 15 minutes (*p = 0.04), and to 84.2 ± 29.9 after 20 minutes (*p = 0.02) of LAD occlusion (Figure 2).

### Ischemic myocardium

**Figure 3:**
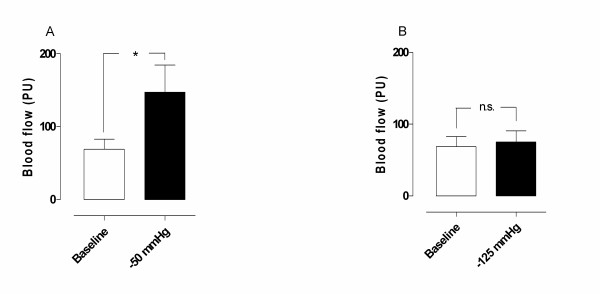
Microvascular blood flow measured using laser Doppler velocimetry in ischemic myocardium exposed to topical negative pressures of A) -50 mmHg, and B) -125 mmHg. The measurements were performed in six pigs at a depth at 1–2 mm in the myocardium. Significance was defined as *p < 0.05, **p < 0.01, ***p < 0.001 and p > 0.05 (not significant, n.s.). Values are presented as means ± the standard error on the mean (SEM).

A topical negative pressure of -50 mmHg induced an immediate significant increase in microvascular blood flow in ischemic myocardium (from 69.0 ± 5.5 PU before, to 147.0 ± 9.1 PU after TNP application, * *p *= 0.04) (Figure 3A). A TNP of -125 mmHg did not result in any significantly change in microvascular blood flow (69.0 ± 5.5 PU before, and 75.0 ± 5.9 PU after, *p *= 0.68) (Figure 3B).

### Conclusion

Post-sternotomy mediastinitis after CABG is a serious and potentially life-threatening complication, with a high mortality rate and substantial morbidity[12,14,29]. Recently, the use of TNP has gained acceptance in the treatment of post-sternotomy mediastinitis, and is today the standard mode of treatment in many cardiac surgery centers [4,5,17,18]. Before the introduction of TNP therapy, the risk of late death in patients suffering from mediastinitis after cardiac surgery was reported to be two to three times higher than that of patients without mediastinitis[12,14,29]. Milano and collages have suggested that mediastinitis may cause negative long-terms effects on several organs such as the heart and kidneys [13]. Theoretically, a massive immunological response during a prolonged period of infection may cause adverse effects on by-pass grafts. In those studies, reporting poor long-term survival after mediastinitis, several conventional wound healing techniques were used (closed irrigation, delayed wound closure, or reconstructing with omentum or pectoral flaps). Interestingly, Sjogren and coworkers, found no difference in long-term survival between CABG patients with TNP-treated mediastinitis and CABG patients without mediastinitis [17]. During the treatment of post-sternotomy mediastinitis, the TNP is in direct contact with the heart, which is exposed through the diastase of the sternotomy. These patients may therefore have developed increased coronary collateral blood vessels during TNP, and may be better prepared when bypass grafts fail to work. We have indeed observed in patients treated with TNP, that richly vascularized granulation tissue develops over the heart within 7–8 days. It may well be that the stimulation of blood flow and the development of collateral blood vessels resulting from TNP in part account for the reduced long-term mortality in patients treated in this way.

We have previously shown that TNP induces an increase in the blood flow of the peristernal soft tissue (i.e. skeletal muscular and subcutaneous tissue), and also that the change is related to local effects, since the blood flow at a distance of 4.5 cm from the wound edge was not affected by the negative pressure[8]. The blood flow increased with elevated subatmospheric pressure in both subcutaneous and skeletal muscular tissue[8]. When the area under the flow-distance curve was analyzed, covering 0.5 to 4.5 cm from the wound edge, a maximal net increase in blood flow was observed at -75 and -100 mmHg, in muscular tissue. Interestingly, a difference in the profiles of the blood flow responses was observed between the subcutaneous and the muscular tissue. The distance from the wound edge to the point at which the blood flow increased was shorter in muscular tissue than in subcutaneous tissue[8]. This may indicate that pressure is transduced differently in a soft and in a dense tissue, and a less dense tissue collapses more easily when affected by pressure. In the immediate proximity of the wound edge, a zone of relative hypoperfusion was observed. This zone was larger at high negative pressures and was especially prominent in subcutaneous tissue. The size of the hypoperfused zone depended on the pressure applied, and increased with increasing negative pressure. In summary, the changes in the peristernal wound blood flow caused by TNP vary with the distance from the wound edge. A few centimeters away from the wound edge, the blood flow increased when subatmospheric pressure was applied. Conversely, in the immediate proximity of the wound, the negative pressure induced relative hypoperfusion[8].

We have recently shown that a myocardial TNP of -50 mmHg significantly increases microvascular blood flow in the underlying myocardium, at a depth of 6–8 mm in the heart muscle, i.e. the middle of the myocardial ventricular wall, in both normal, ischemic, and reperfused porcine myocardium[27]. We have also demonstrated that TNP levels between -75 mmHg and -150 mmHg, applied to the myocardium, do not alter myocardial microvascular blood flow, also at depths at 6–8 mm down into the myocardium[30]. In skeletal muscle and subcutaneous tissue, application of different negative pressure levels causes different changes in microvascular blood flow[8]. When the negative pressure exceeds a specific level it seems to constringe the vessels in subcutaneous tissue and skeletal muscle and a decrease in microvascular blood flow close to the vacuum source, i.e. a zone of hypoperfusion, is seen[8]. The hypoperfusion zone seen in subcutaneous tissue was greater then the one seen in skeletal muscle at same pressure levels[8]. However, when applying TNP to the myocardium, a significant, long-standing zone of hypoperfusion could theoretically cause ischemia, and negative effects on the myocardium. The current study was designed to determine whether a zone of relative hypoperfusion was produced during myocardial TNP, as seen in subcutaneous and muscular tissue[8]. In the present study we show that, a TNP of -50 mmHg applied over the LAD region significantly increases the microvascular blood flow in normal myocardium, while a TNP of -125 mmHg did not significantly alter the microvascular blood flow. This indicates that no hypoperfusion is produced in normal myocardium during TNP. In ischemic myocardium, application of a TNP of -50 mmHg over the LAD region resulted in a significant increase in microvascular blood flow. However, a pressure of -125 mmHg did not induce any significant change in microvascular blood flow in the ischemic myocardium. Consequently, no hypoperfusion could be observed during myocardial TNP in neither normal nor ischemic myocardium. The absence of hypoperfusion might be explained by the higher density of myocardium than skeletal muscle and subcutaneous tissue.

Among patients with ischemic heart disease, some are not suitable for current revascularization procedures such as percutaneous coronary interventions (PCI) and CABG, because of extensive and technically problematic coronary lesions, i.e. refractory angina pectoris. The natural response to myocardial ischemia is neovascularization. This angiogenic process is a physiological attempt to limit myocardial ischemia. Therapeutic angiogenesis, wherein exogenous growth factors are administered to ischemic tissue to enhance reperfusion, has been investigated as a potential form of treatment for patients with advanced coronary artery disease as an alternative to conventional treatment such as PCI and CABG. VEGF proteins have been shown to play a key role in the modulation of angiogenesis and vascular growth [20,21]. Interestingly, TNP produces a mechanical shear stress that is known to activate endogenous VEGF[25,26,31-33].

## Conclusion

In conclusion, no hypoperfusion could be observed in the epicardium in neither normal nor ischemic myocardium during myocardial TNP. Myocardial TNP may in the future, constitute an alternative therapeutic intervention to stimulate blood flow in the failing myocardium in patients with ischemic heart disease.

## List of abbreviations used

CABG Coronary Artery Bypass Grafting

LAD Left Anterior Descending Artery

PCI Percutaneous Coronary Interventions

PU Perfusion Units

TNP Topical Negative Pressure

VEGF Vascular Endothelial Growth Factor

## Competing interests

The author(s) declare that they have no competing interests.

## Authors' contributions

SL and RI carried out the animal studies. SL and MM carried out the acquisition, analysis, and the interpretation of the data. SL wrote the manuscript. RI, MM, and SL made substantial contribution to concept and design of the study.
